# Heat Stress Prevention and Management in the Construction Industry: A Gap Analysis

**DOI:** 10.5334/aogh.4833

**Published:** 2025-10-31

**Authors:** Muinat Abolore Idris, Kristina D. Mena, Christine Markham, William B. Perkison

**Affiliations:** 1 Department of Environmental and Occupational Health Sciences, University of Texas Health Science Center at Houston School of Public Health, Houston, Texas, USA; 2 Department of Human Promotion and Behavioral Sciences, University of Texas Health Science Center at Houston School of Public Health, Houston, Texas, USA

**Keywords:** heat-related conditions, heat stress programs/interventions, well-being, heat stress safety and health practices, gap analysis

## Abstract

*Background:* Despite decades of efforts to prevent heat stress, it remains a major health risk to workers in the construction industry. This health risk has been exacerbated by the rising ambient temperatures from climate change, as well as the increased susceptibility to heat stress in an aging workforce.

*Objective:* To develop a tailored occupational safety management framework for heat stress prevention in construction, advancing workers’ health, safety, and well-being.

*Methods:* We built on the findings of a systematic review and analyzed a large industrial company’s current heat stress prevention program through stakeholder engagement, evidence-based practices, and gap analysis. We reviewed the American Conference of Governmental Industrial Hygienists Threshold Limit Values, the International Standard Organization, and country-specific standards and used the National Institute for Occupational Safety and Health (NIOSH) recommended criteria as a benchmark. Gaps were identified by comparing existing measures to best practices.

*Findings:* Most Wet-bulb Globe Temperature (WBGT) measurements (24.4°C–53.7°C) from the systematic review were collected over short durations without accounting for rest breaks, potentially underestimating or overestimating workers’ heat stress and heat-related injuries/illnesses (HRIs) risks. Rest breaks did not follow the WBGT values, which is crucial for mitigating heat stress. Water consumption from the systematic review was below the NIOSH recommendations, with no electrolytes provided. Working 10 h/day, 13 days/two weeks, increases fatigue levels, significantly impacting workers’ sleep quality and HRI risks.

*Conclusion:* Preventing heat stress, addressing heat stress management gaps, and advancing construction workers’ health, safety, and well-being require stakeholder involvement at all levels.

## Introduction

**Figure 1:**
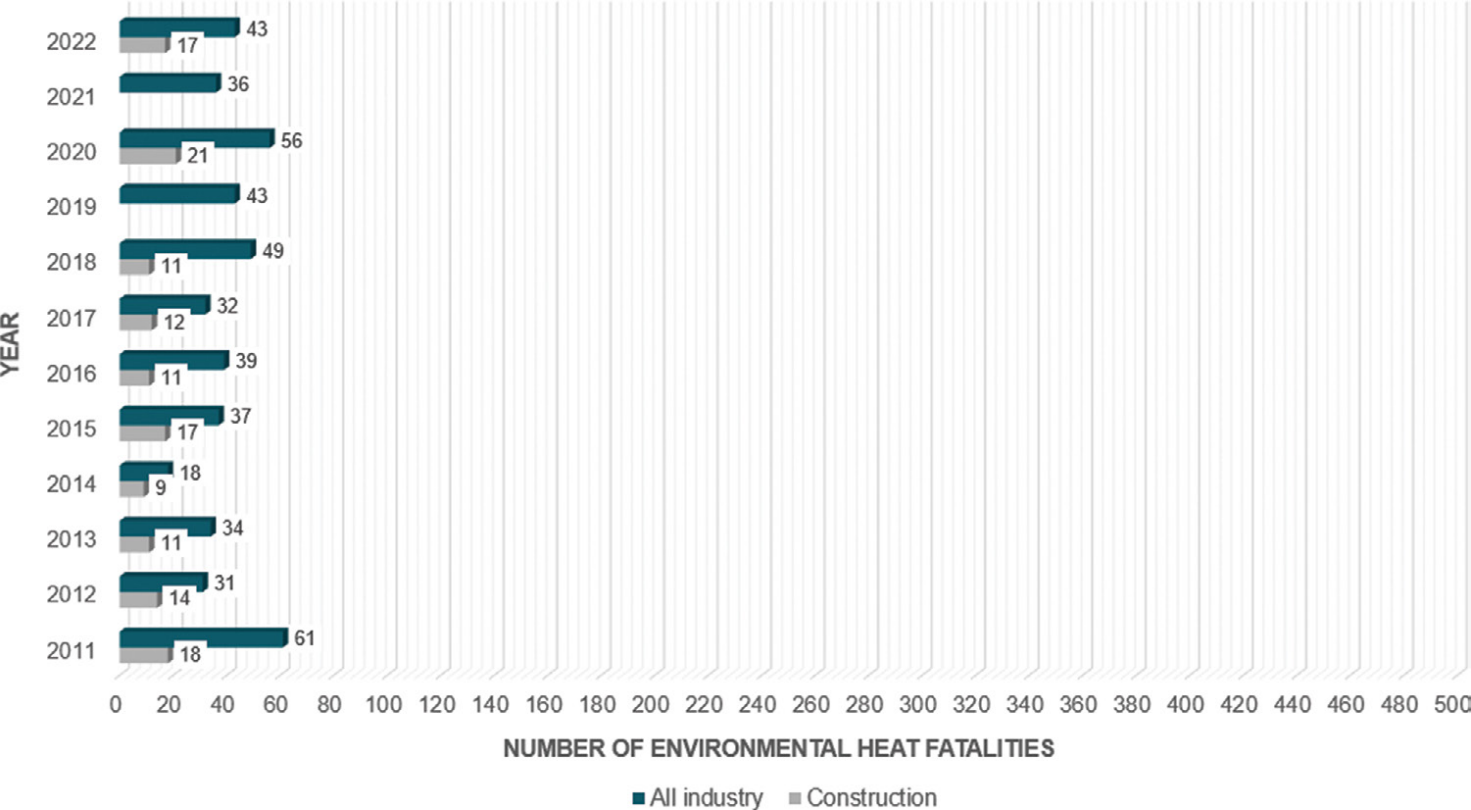
Reported heat-related fatalities among U.S. construction workers, 2011–2022.

Heat stress is a globally pressing environmental and occupational health concern due to occupational heat exposure among outdoor workers. It is characterized by the inability of the human body to dissipate heat effectively, leading to heat-related injuries and illnesses (HRIs) that can negatively impact workers’ health, significantly threaten well-being and quality of life (QOL), and even lead to deaths. Occupational heat exposure reduction awareness and prevention of heat stress and heat-related conditions have been in existence for several decades. Agencies and organizations such as the National Institute for Occupational Safety and Health (NIOSH), the International Standard Organization (ISO), the American Conference of Governmental Industrial Hygienists (ACGIH), the Occupational Safety and Health Administration (OSHA), and the American Industrial Hygiene Association have developed occupational heat exposure guidelines or standards to prevent heat stress. Countries such as China, Saudi Arabia, and Hong Kong have also developed specific heat standards. Despite these efforts, heat stress remains a significant concern to outdoor workers, including construction workers, due to the projected increasing average ambient temperatures and an aging workforce. For example, between 2011 and 2022 [1], 141 U.S. construction workers died from environmental heat exposure, with 21 deaths in 2020 alone, nearly double the number in 2013 (*n* = 11) (Figure 1). Additionally, the percentage of U.S. construction workers aged 55 and above nearly doubled from 11.5% in 2003 to 22.7% in 2020 [2]. This reflects the U.S. aging population and the overall employed aged 55 and over, which increased from 15.4% to 23.9% during this period [2] and is expected to increase by 2030 through 2050 [3–5].

Aside from the developed heat guidelines by these agencies, organizations, and each country, studies show that several heat stress interventions and prevention programs have been developed and implemented to prevent heat stress and heat-related conditions. Our previous systematic review [6] found that interventions, including acclimatization, environmental monitoring, hydration practices, rest breaks, cooling practices, and training, have been implemented, but that numerous interventions did not achieve a complete reduction in heat stress illness for workers. Despite all the growing awareness of the dangers associated with occupational heat exposure, heat stress remains a threat to outdoor workers’ health, safety, and well-being, indicating the need for a gap analysis to identify lapses and understand the gaps in preventing heat stress.

This study focuses on construction workers to assess the gap between current occupational recommended guidelines and implemented interventions. We conducted a gap analysis to develop an occupational safety management framework tailored to heat stress prevention for the natural gas construction work environment to advance workers’ health, safety, and well-being. In developing the management framework, the gap analysis of the effectiveness of current heat stress mitigation strategies was identified through stakeholder engagement, evidence-based practices, and gap analysis methodology. Although gap analysis is rarely used in the occupational safety and health sector, it has been a helpful tool in healthcare to evaluate current practices and desired performance. According to the Agency for Healthcare Research and Quality [7], (p. 305), gap analysis helps improve hospital quality and safety by identifying the differences between the current clinical and recommended practices. It can identify the gaps by systematically comparing current approaches to evidence-based standards/guidelines [8]. The gap analysis conducted in this study evaluated the critical gaps and challenges identified in our previous qualitative and systematic review studies in preventing and managing heat stress among construction workers to answer a research question. “How can workers’ health, safety, and well-being in relation to heat stress be improved using the heat stress interventions?”

## Methods

A gap analysis integrating stakeholders’ engagement was conducted to evaluate current heat stress standards, including NIOSH recommendations, ACGIH TLV guidelines, and ISO 7243, as well as multiple country practices, with different identified heat stress prevention approaches. The analysis was intended to identify areas for further research in improving and advancing the health, safety, and well-being of construction workers at risk of heat stress.

### Study procedures

#### Brief description of previous studies

For the qualitative study, [9] 21 stakeholders participated in semi-structured key informant interviews and six focus group sessions. The key informant participants were two safety officers, and the focus groups comprised 19 natural gas construction workers. These participants worked in the morning for 10-h shifts for 13 consecutive days with one day off. The collected data were analyzed using framework analysis and coded using an inductive coding approach and thematic analysis to identify themes. The systematic review [6] followed the PRISMA procedures and a PICO framework. The initial search yielded 3625 studies from four databases: PubMed, Embase, CINAHL Plus, and Web of Science. Following the review for relevance of the topic, removal of duplicate studies, review of titles and abstracts, and full-text review, 36 studies met the inclusion criteria. Of these, 12 studies assessed heat stress prevention programs/interventions among construction workers across the continents, which were analyzed in this study.

#### Gap analysis

**Figure 2:**
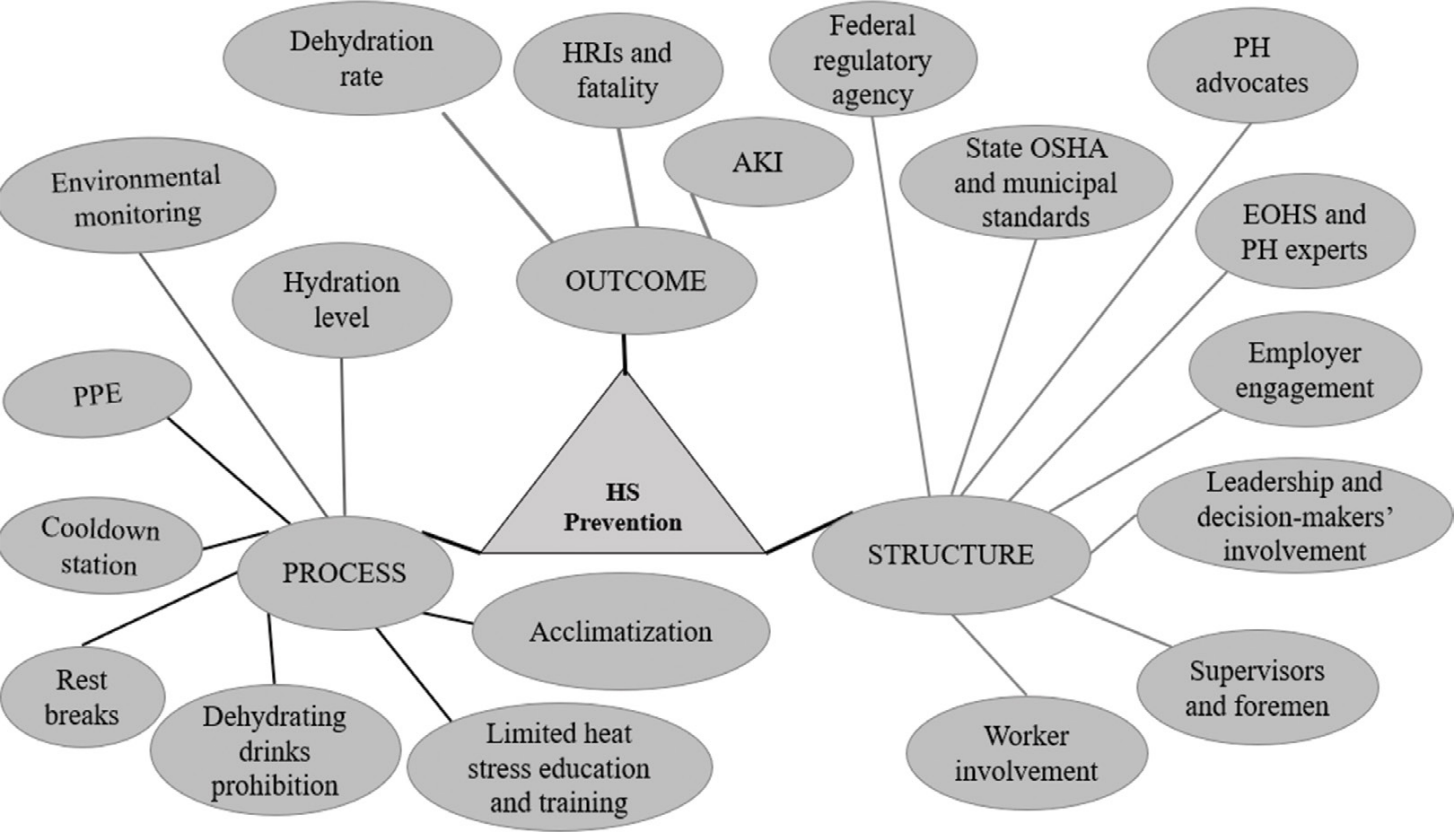
Heat stress prevention conceptual map to advance construction workers’ health, safety, and well-being. *Note*: PH: public health; EOHS: environmental and occupational health and safety; HS: heat stress; PPE: personal protective equipment (cooling vests); WBGT: Wet-bulb Globe Temperature; AKI: acute kidney injury; HRIs: heat-related injury and illness.

Building on the previous findings, we identified the current heat stress safety and health practices situation and the different approaches towards heat stress prevention in advancing construction workers’ health, safety, and well-being. This defined our important metric of interest: “advancing workers’ health, safety, and well-being when working in a hot environment/exposed to occupational heat/heat stress following the ongoing increasing temperatures and the projected aging workforce.” We analyzed gaps between what was observed and identified and what should be done to reduce heat stress risks, prevent heat stress, and advance workers’ health, safety, and well-being. We classified the gaps using evidence-based items identified from the systematic review [6] and subjective evaluation from the qualitative study [9]. We organized the items using the Donabedian domains (structure, process, and outcomes) [10] and developed a conceptual map (Figure 2) to inform the development of the Heat Stress Safety and Health Practice (HSSHP) Gap Analysis. Classifying the problem into domains allowed us to focus on the issues and helped guide the gap analysis. The conceptual map was used to identify the essential components and visually depict conceptual relationships [11] for the researchers to discuss and better understand the interrelationships among heat stress, heat stress prevention programs/interventions, and workers’ health, safety, and well-being. We defined the best practices by reviewing the appropriate legal and regulatory expectations, which were (i) ACGIH TLV [12], (ii) ISO 7243 [13], and (iii) multiple countries’ occupational safety and health regulations for heat stress/exposure. The ACGIH TLV Wet-bulb Globe Temperature (WBGT) is calculated as follows:


$$\text{WBGT}=0.7\;\times \;T_{\text{nwb}}\;+\;0.2\;\times \;T_{\text{g}}\;+\;0.1\;\times \;T_{\text{db}},$$ 1

where:

*T*_nwb_ = measured natural wet bulb temperature

*T*_g_ = measured globe temperature

*T*_db_ = measures dry-bulb (air) temperature

NIOSH’s updated criteria [14] for heat exposure were used as the benchmark. The identified measures were compared with the best practice strategies/standards and the benchmark to identify gaps.

An occupational safety management framework for heat stress was developed based on the strengths, weaknesses, opportunities, and threats analysis [15] identified from the gaps. This analysis allowed us to explore perspectives from various stakeholders to guide the development of heat stress prevention strategies.

## Ethical Considerations

This study was approved by the UTHealth Houston Institutional Review Board Committee (HSC-SPH-23-0469).

## Results

### I. Stakeholders’ perception: qualitative assessment of natural gas construction workers’ overview

The qualitative study with natural gas construction site employees and managers identified three themes (heat stress intervention context, factors influencing workers’ safety, and intervention outcome and worker well-being), as well as eight sub-themes. Working under pressure and self-pressure, co-workers’ work practices, unsupportive bosses, generational differences, ignoring co-workers’ suggestions, abusive use or abandoning employer-provided resources (like not drinking water, overdoing water, and overdoing electrolytes without water), and work schedules and shift hours were some identified barriers impacting workers’ safety. In contrast, employee preparedness the night before, provision of resources by employers at no cost with easy accessibility, using a hydration log to monitor and audit workers’ hydration status, coupled with real-time communication strategies to increase workers’ heat level alert, were the key identified facilitators [9].

### II. Evidence-based practices from the systematic review: identified heat stress interventions among construction workers

Six key characteristics (i.e., heat stress interventions/programs) were identified from the 12 studies that assessed the construction industry [6]. Environmental monitoring, which includes measuring parameters like WBGT, heat index, and thermal work limit (*n* = 11), followed by rest breaks/lunch breaks (*n* = 10) and hydration practices (*n* = 9), were the most identified interventions (Table 1). The use of personal protective equipment (cooling gear/vest = 4) as a cooling practice, which was mostly worn for a short duration of time (between 15 min and 2:15 h), especially during rest breaks for 15 min and 30 min, was also identified. The least identified intervention was training (*n* = 1).

**Table 1:** Heat stress programs/interventions within the construction sector as derived from a systematic review.

IDENTIFIED CHARACTERISTICS/ INTERVENTION	AUTHORS (*N* = 12)
Environmental monitoring	Ahmed et al. [16]; Al-Bouwarthan et al. [17]; Ashtekar et al. [18]; Chan et al. [19]; El-Shafei et al. [20]; Farshad et al. [21]; Montazer et al. [22]; Ueno et al. [23]; Yang & Chan [24]; Yasmeen et al. [25]; Zhao et al. [26] (*n* = 11)
Acclimatization	Al-Bouwarthan et al. [17]; Ashtekar et al. [18]; El-Shafei et al. [20]; Ueno et al. [23]; Yang & Chan [24]; Yasmeen et al. [25]; Zhao et al. [26] (*n* = 7)
Hydration practices (water)	Al-Bouwarthan et al. [17]; Ashtekar et al. [18]; El-Shafei et al. [20]; Farshad et al. [21]; Montazer et al. [22]; Ueno et al. [23]; Yang & Chan [24]; Yasmeen et al. [25]; Zhao et al. [26] (*n* = 9)
Rest breaks/lunch breaks	Ahmed et al. [16]; Al-Bouwarthan et al. [17]; Ashtekar et al. [18]; Chan et al. [19]; El-Shafei et al. [20]; Montazer et al. [22]; Ueno et al. [23]; Yang & Chan [24]; Yasmeen et al. [25]; Zhao et al. [26] (*n* = 10)
Cooling practice measures (PPE)	Ashtekar et al. [18]; Chan et al. [19]; Yang & Chan [24]; Zhao et al. [26] (*n* = 4)
Training	El-Shafei et al. [20] (*n* = 1)

### III. HSSHP gap analysis

In developing the conceptual map (Figure 2), 41 evidence-based and stakeholder practices were identified and organized using the Donabedian domains of structure, process, and outcomes. The evidence was grouped into 19 concepts and further categorized into eight structures, eight processes, and three outcome concepts. The qualitative study and the systematic review findings were then compared to the benchmark (NIOSH criteria), the ACGIH TLV, the ISO 7243, and each country’s regulations. Identified gaps include environmental monitoring, rest breaks, hydration practices, training, and work–life balance.

*Environmental monitoring:* Comparing the measured WBGT values from the systematic review to the ACGIH TLV, ISO 7243, NIOSH criteria, and country-specific regulations, most of the measured WBGT values were collected for shorter durations (15 min, 30 min, and 3 h). Some were collected for the entire work shift, excluding workers’ break periods, with only one study including break periods. The measured WBGT values from some of these studies might have either been underestimating or overestimating worker exposures, potentially leading to (i) increased heat stress risk, (ii) implementation of unnecessary control or excessive preventive measures, and (iii) waste of resources. For instance, when WBGT is collected for periods less than 8 h, the collected values should be interpreted as a time-weighted average (TWA) and not the regular TLV (i.e., 8-h shift). None of the studies that collected WBGT for less than 8 h reported their findings as TWA. Accurate WBGT measurement and interpretation are essential for proper heat stress evaluation and prevention. Notably, this workforce works for approximately 8–12 h daily.

*Rest breaks:* These were not taken following the measured WBGT values throughout the entire work shift. Though participants from the qualitative study [9] reported taking breaks every hour when the temperature is ≥ 100° Fahrenheit “… When … the temperature hits like a 100 or 100 plus, they [employer] want you to keep taking breaks every other hour, …” [Painter ID: 6] (p. 7), but the break duration was not stated*.* These hourly breaks and the permitted 10–15 min rest breaks with the 30–90 min lunch breaks reported from both the qualitative study and systematic review may not sufficiently protect workers from heat stress. According to the NIOSH heat stress work/rest schedules, a worker performing heavy work in (104°F) temperatures is expected to work for 20 min and rest for 40 min, as compared to a worker performing moderate work in the same temperatures who is expected to work for 30 min and rest for 30 min. Taking rest breaks per measured WBGT values helps in preventing heat stress and heat-related conditions, as WBGT is the key factor in determining heat stress risks associated with workers working in adverse heat conditions. Also, per NIOSH’s recommended criteria [27] (benchmark), workers’ work/rest schedules should be based on the physical job demand, air temperature, adjustment from direct sunlight and humidity, and WBGT to prevent heat-related conditions.

*Hydration practices:* Comparing findings from the systematic review to the benchmark, most hydration practices were inadequate to keep workers hydrated. Workers reported consuming below the NIOSH recommended frequency (8 ounces/0.23 liters (L) of water every 15–20 min) and the expected total daily volume of 5.25–7.00 L of water for an 8-h shift. Also, none of the 12 construction workers’ studies in the systematic review reported the employer provision or worker consumption of electrolyte beverages, which NIOSH recommends for workers exposed to heat for more than 2 h. While participants in the qualitative study reported the availability and easy accessibility of water and electrolytes, the volume consumed and their self-reported underutilization of these provisions indicate the need to better understand this limited use. A quantitative measurement of the worker’s fluid intake was beyond the study’s scope.

*Training:* Findings from both the systematic review and qualitative study indicate workers are generally provided with limited or insufficient training. Though workers from the qualitative study reported receiving training, the heat stress training is informally provided alongside other health and safety training during the daily safety toolbox talks and weekly meetings. Only one study from the systematic review described training by assessing a health education program intervention on workers’ knowledge and behavior towards exertional heat illness.

*Work–life balance:* Though this was not part of the benchmark criteria, the issue was reported in both the qualitative and systematic review findings. Based on the findings from the qualitative study, working 10 h per day, 13 days per two weeks, with just one day off, could significantly impact workers’ sleep quality, which may further affect well-being.

### IV. Occupational safety management framework

**Figure 3:**
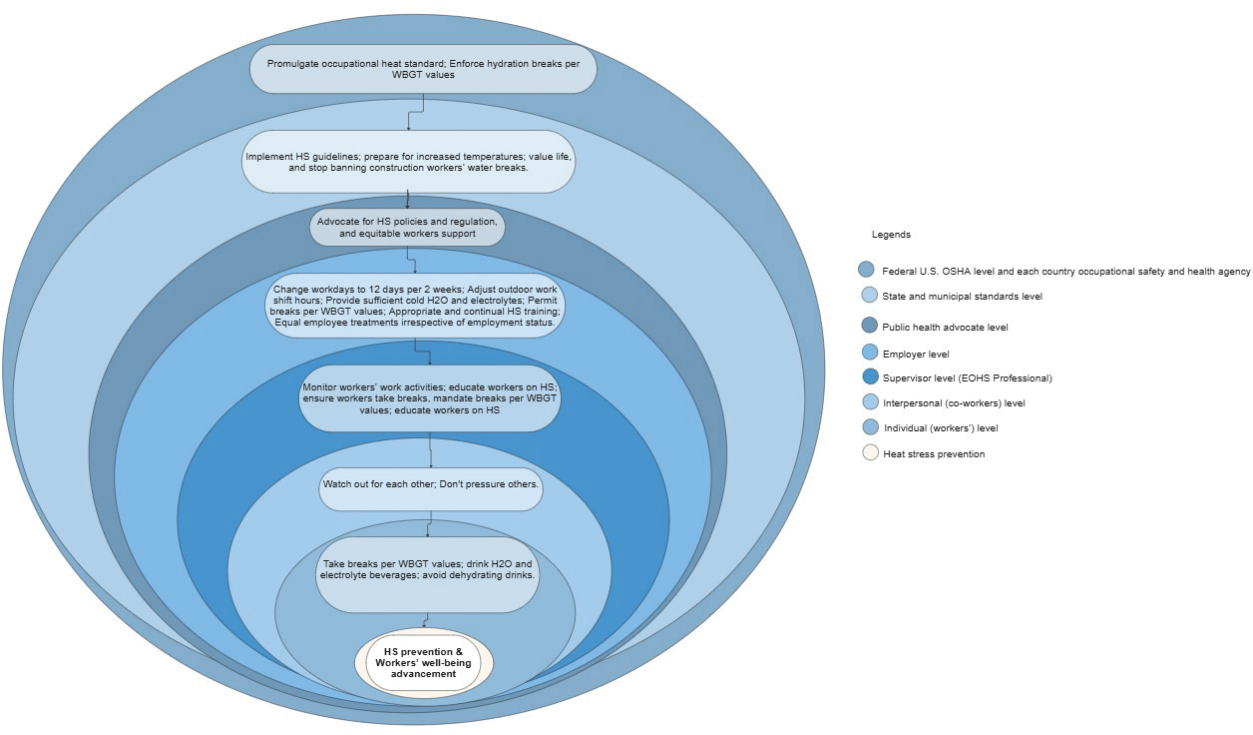
Heat stress safety management framework.

Preventing heat stress and improving workers’ health, safety, and well-being require collaborative efforts. With the rising ambient temperatures, an aging workforce, and increasing heat-related conditions and fatalities, personnel at every level of an organization must adhere to their responsibilities in promoting workers’ well-being. Figure 3 shows a heat stress occupational safety management framework outlining each stakeholder’s responsibilities for preventing heat stress and advancing workers’ health, safety, and well-being.

## Discussion

Prevention of heat stress and heat-related conditions is a complex national and global occupational health and safety issue. It is of concern to public health experts, environmental health specialists, occupational health and safety professionals, and vulnerable workers, including construction workers. Collecting WBGT without accurately following the guidelines, taking rest breaks without considering the daily measured WBGT values, not drinking enough water with electrolytes daily to keep workers hydrated, and a lack of formal heat stress training were the key identified gaps in preventing heat-related conditions and advancing workers’ health, safety, and well-being.

### I. Environmental monitoring

Collecting WBGT for less than 1 h may not capture workers’ exact heat exposure and may underrepresent the exposure, as WBGT value collection requires a stable period of measurement. WBGT measurement requires a representative period of about 1 h [13], as it takes the black globe and the natural wet bulb thermometer of the WBGT at least 20 min to come to a steady state with the environment [28, 29]. Unfortunately, some studies identified in the systematic review collected WBGT values for 15 or 30 min, which likely might have underestimated heat exposure, increased heat stress risk, and workers’ vulnerability to heat-related conditions. Additionally, collecting WBGT for less than 8 h or full shifts, excluding WBGT data for workers’ break periods, and calculating the WBGT using Equation (1) may either underestimate or overestimate worker heat exposure. This approach is inconsistent with the ACGIH TLV and ISO 7243 WBGT guidelines, which were developed with the assumption of 8-h workdays in a 5-day workweek. Irrespective of the heat exposure and WBGT guidelines in use, whenever the work environment is monitored for less than or over an 8-h shift, the collected WBGT values should be adjusted, analyzed, and reported as TLV–TWA and not the regular TLV that was developed with the assumption of an 8-h shift.

Also, based on the ACGIH TLV, WBGT can only be measured for 3 h if the exposure is constant and the metabolic work rate is steady, which requires some calculations. Due to the nature of construction workers’ jobs, their metabolic rate is not always constant, and it can vary depending on the work category, ranging from light to very heavy. When the exposure or the metabolic work rate varies, the recommended TWA for WBGT and metabolic work rate over a period of about 1 h is recommended [30]. This is also supported by the Canadian Center for Occupational Health and Safety [28]; when WBGT is collected for less than 8 h or when the thermal conditions of the workplace fluctuate widely, the WBGT values need to be calculated as TWA using Equation (2). Calculating collected WBGT values this way helps to accurately estimate workers’ heat exposure and avoid underestimating or overestimating the environmental heat and workers’ heat stress risk level, which could either negatively impact workers’ health, safety, and well-being or result in the waste of resources through the implementation of unnecessary control measures that may hinder workers’ performance.

Consistent with the ACGIH TLV guideline, ISO 7243 [13] requires a measurement of WBGT over a representative period of about an hour. ISO 7243 recommended that if the work over a day is divided into distinctly different types or categories, it may be necessary to make separate measurements and assessments of the different types of work. For example, when there is mainly light work in the morning and heavy work in the afternoon, or when the WBGT values are significantly different for periods of over an hour. For instance, for a 10-h shift worker with 2 h of light work in the morning, 6 h moderate work in the midday, and 2 h heavy work in the afternoon, the WBGT for each condition should first be calculated using Equation (1), resulting in three WBGT values. Then, the calculated values will be used in the below equation to compute a single WBGT TWA value estimating workers’ 10-h total exposure:


$$\text{TWA WBGT }=\;\frac{{\text{WBGT}}_{1}\;\times \;t_{1}\;+\;{\text{WBGT}}_{2}\;\times \;t_{2}\;+\;....\;+\;{\text{WBGT}}_{n}\;\times \;t_{n}}{t_{1}+t_{2}+\;....\;+t_{n}}.$$ 2

where:

WBGT_1_, WBGT_2_, WBGT*_n_* = measured or calculated WBGTs

*t*_1_, *t*_2_, *t_n_* = elapsed time spent based on the WBGT_1_, WBGT_2_, WBGT*_n_*, respectively.

### II. Rest breaks

Rest breaks are important in heat stress prevention; however, not considering WBGT values when implementing rest breaks is a critical gap. Scheduled breaks without considering WBGT can leave workers exposed to unsafe working conditions. An agricultural study found that even with a scheduled 75% work and 25% rest regimen, workers still spent over 40% of their working time beyond the recommended work/rest guidelines [31]. This supports the importance of utilizing the WBGT values to determine the suitable period, time, and rest break durations. Also, with the reported WBGT values from our systematic review [6], ranging from 21.7°C (71.06°F) to 53.7°C (128.66°F), both the permitted and scheduled rest breaks of approximately 10–15 min with lunch breaks of 30–90 min are insufficient in mitigating heat stress and advancing workers’ health, safety, and well-being. We strongly recommend the use of the daily measured WBGT values to determine the suitable rest break periods for workers over the full shift. Studies show that break duration and frequency, along with the availability of shade, are crucial factors in reducing heat stress [32]. However, future research should investigate the relationship between WBGT values and the rest break frequency with duration to better prevent outdoor workers from heat stress.

### III. Hydration practices

Based on the NIOSH recommended hourly [0.70 L–0.94 L (24–32 ounces)] and the calculated 8-h daily shift (5.6–7.52 L), the expected volume of water to consume, the consumption of an average of 2.6 L of water daily [23], with an average of 5.9 ± 1.0 L fluid intake [17] for a daily work shift of approximately 8–12 h is low and may be insufficient to keep workers hydrated. This presents a significant gap in mitigating heat stress and heat-related condition risks. Daily consumption of 2.6 L for an 8-h shift indicates workers consume approximately 0.32 L (11.83 ounces) per hour, which is below the recommended volume and may compromise workers’ safety by increasing their heat-related condition risks. Additionally, knowing that workers were not provided with electrolyte supplements from the systematic review to support their body systems, we strongly recommend that employers offer electrolyte beverages at no cost, as the loss of electrolytes can lead to muscle cramps and other dangerous health problems. The low intake of water, combined with a lack of electrolyte supplements, increases workers’ risk for HRIs.

### IV. Work–life balance

This is not included in the NIOSH guideline that served as the benchmark for this gap analysis. However, evaluating this is crucial considering construction workers’ average 10-h daily shifts for 13 consecutive days with one day off. This schedule can significantly impact workers’ physical and mental health. It has been well established that cumulative long work hours with limited rest potentially lead to several health and well-being threats, including increased fatigue, musculoskeletal disorders, cognitive impairment, cardiovascular disease risk, and reduced work performance [33–40], which can significantly reduce overall QOL. As recommended in our previous systematic review, we emphasize the re-evaluation of construction workers’ 10-h work shifts in hot environments to address this gap and mitigate heat stress risk to advance workers’ health, safety, and well-being. Additionally, addressing this gap will significantly improve workers’ productivity, which is very beneficial to every employer, as several studies have shown that extreme heat exposure reduces workers’ productivity.

### V. Heat stress safety management framework

Heat stress prevention and mitigation management is a complex environmental and occupational health issue that requires coordinated efforts from stakeholders across the levels described in the designed framework to achieve the goal of “heat stress prevention and workers’ well-being advancement.” Facilitating a strong safety culture and advancing worker well-being depends on effective stakeholder engagement—an established evidence-based approach that is well supported in the healthcare sector. Studies show that interprofessional collaboration is a crucial healthcare reform strategy [41, 42], and collective leadership has proven to improve healthcare staff’s well-being by reducing stress [43]. This finding is supported by a systematic review that found that stakeholder input and involvement are essential in designing effective dietetic services that enhance patient engagement [44]. These examples accentuate the importance of multi-level collaboration in addressing heat stress, promoting safety, and advancing health and well-being. Also, the effective implementation of a lifestyle intervention requires tailored communication at all organizational levels, with clear intervention goals [45]. The levels in the framework were grouped into the following three categories:

#### A. Policy/decision-makers’ role in shaping the future of workers’ safety, health, and well-being

This section addresses each country’s occupational safety and health (OSH) agency, the U.S. federal OSHA, and state and municipal government responsibilities. It is imperative that OSHA, along with each country’s OSH agency, disseminate and enforce standardized occupational heat regulation, including mandatory hydration breaks based on daily WBGT values—the reliable and comprehensive heat stress index. This regulation/standard would help prevent heat stress, reduce HRI risk, improve safety, enhance workers’ health and well-being, and support productivity.

While countries, including Hong Kong, Australia, Saudi Arabia, Qatar, etc., have established regulations, the U.S. OSHA currently relies on the General Duty Clauses, and states including California, Washington, and six other states, have enforceable standards. The need for U.S. federal action is urgent, particularly as some states roll back water break requirements, stripping workers of their most basic hydration rights and blocking cities and counties’ local authorities from protecting workers. For instance, in 2024, states including Florida (83.0°F), Texas (80.8°F), Louisiana (79.2°F), Hawaii (74.1°F), and Mississippi (77.6°F) were the top five hottest states with the highest annual average temperatures reflecting the growing climate change impact. That same year, Florida, Texas, and Louisiana experienced extremely high summer temperatures, increasing HRI risks among construction workers. Following the ongoing increase in temperatures, outdoor workers in these states, particularly those working in agriculture and construction sectors, will remain increasingly vulnerable to HRI risks, and their overall well-being will be drastically impacted.

State and municipal governments must take proactive measures by adopting comprehensive heat stress guidelines, halting the rollback of worker protections, like water break bans, and prioritizing human life over deregulation. With heat-related productivity loss costing approximately $100 billion annually, which could double by 2030, outdoor industries (agriculture and construction) have the steepest losses [46]. Establishing and adopting robust heat stress guidelines will keep workers safe, productive, and protected from preventable HRIs and fatalities. Also, valuing workers’ lives is essential, as workers’ lives should never be sacrificed for corporate interests, and it is the government’s moral and legal responsibility to ensure that no one dies from preventable HRIs; this can significantly contribute to the economy by improving workers’ productivity. Policies, including the Texas House Bill 2127, which limits municipal authority to mandate water breaks, can have implications, endangering workers and contributing to increased productivity loss. Studies found that an increase in the number of high-temperature days leads to reduced labor productivity, highlighting the economic significance of climate impacts and the need for developing enhanced adaptation policies to mitigate future climate-related economic losses [47]. Therefore, adopting evidence-based heat stress guidelines, reinstating workers’ hydration protections, and funding workplace programs to prevent HRIs and protect workers’ health and well-being at the state and municipal levels would also improve economic sustainability.

#### B. Implementers’ role in executing heat stress prevention regulations

This section includes the public health advocates, employers, and supervisors.

Public health advocates need to continue to advocate for heat stress regulations to protect these disproportionately affected workers. With their efforts focused on establishing national heat stress standards, ensuring equitable workplace protections and support, and opposing legislation that weakens worker rights and safety regulations, heat stress long-term solutions would be promoted, and vulnerable outdoor workers’ well-being will be advanced.

Workplace protection should be a fundamental human right that should not be determined by job classification or economic status [48, 49]. Advocacy, including workers’ equity support, pushing for hydration breaks, work–rest cycles based on WBGT values, and educational campaigns, would also help to prevent heat stress and advance workers’ safety, health, and well-being. Studies show that successful campaigns in sports are relevant strategies for preventing HRIs [50], and the Columbia Gorge Fruit Growers’ advocacy led Oregon OSHA to create its new heat protection regulations in 2022. By leveraging scientific evidence and partnerships with governments, labor organizations, and communities, public health advocates can influence heat stress policies, promote equitable workplaces and evidence-based solutions to combat heat stress, and promote worker safety, health, and well-being.

Employers must ensure equal protections to all employees—irrespective of employment status (full-time, part-time, seasonal, or temporary)—enforce mandatory rest breaks based on WBGT values, and implement comprehensive heat stress training. Addressing disparities in workplace protection is crucial, and proper training helps to equip workers and supervisors with the essential knowledge required to prevent heat stress and significantly reduce HRI risks. Additionally, re-evaluating and modifying workers’ daily 10–12-h shifts (≥ 60 h/week) and adjusting the work schedules, especially during summer, is crucial to preventing heat stress. This is supported by the International Labor Organization report about the Qatar legislation. The adjustment of Qatar outdoor workers’ working hours in 2022 led to a reduced number of heat-related disorder clinic visits (*n* = 351) in the summer, as compared to prior to the legislation, with 620 in 2021, 1520 in 2020, and 1320 in 2019 [51] heat-related disorder clinic visits. Also, working over 55 h/week can significantly impair workers’ cognitive efficiency and increase injury risks [35, 52]. Therefore, prioritizing workers’ safety would significantly contribute to heat stress prevention and advance health and well-being.

Supervisors are key frontline implementers in preventing heat stress by monitoring workers’ activities, enforcing WBGT based rest breaks, ensuring compliance, and identifying early heat stress signs. Studies found that training supervisors and workers to recognize heat stress is a preventative method [53], and the implementation of training for workers and supervisors about symptom recognition and first aid must be included in a comprehensive HRI prevention program [54].

#### C. Affected/impacted population responsibilities in preventing heat stress

This section includes the interpersonal and individual-level sectors. While leadership and policy play central roles, interpersonal relationships and individual actions are also crucial. A supportive workplace culture—where co-workers watch out for one another and discourage unsafe behaviors including pressuring peers to keep working in extreme heat—is the cornerstone of effective heat stress prevention. When workers feel psychologically safe about speaking up about their concerns, they are more likely to practice the buddy system and follow the heat stress regulations. A scoping review found that psychosocial safety climate directly affects workers’ health, safety, well-being, and performance outcomes [55]. Creating a psychosocial safety climate would foster safety practice adherence, which will advance workers’ health and well-being.

Finally, at the individual level, it is essential that individual workers actively participate by taking rest breaks based on daily WBGT values, staying hydrated by consuming sufficient water and electrolyte beverages, and avoiding the consumption of dehydrating drinks and foods, including soda. Taking rest breaks based on WBGT values is essential, as intense exercise/physical exertion in extreme heat conditions significantly increases core body temperature [56], which puts workers at risk for HRIs and even fatality. Also staying hydrated is not just about drinking water—but maintaining fluid and electrolyte balance. When workers sweat, they lose both water and electrolytes, including sodium, potassium, and magnesium—essential for thermoregulation and overall human body function. Electrolyte imbalance can lead to muscle cramps and other potential health issues, which can impact workers’ health, safety, and well-being.

## Conclusion

Overall, collecting and analyzing WBGT data without accurately following the guidelines, taking rest breaks without considering measured WBGT values, drinking water below the NIOSH recommended frequency and daily volume of water per entire work shift duration, lack of formal heat stress training, and poor work–life balance were the identified gaps in preventing heat stress and heat-related conditions among construction workers. Accurately following guidelines when monitoring work environments via collecting WBGT values, taking rest breaks per measured WBGT values, and ensuring the supply and accessibility of good quality water with electrolyte beverages are highly recommended to avoid overestimating or underestimating workers’ heat exposure, prevent heat stress, and advance workers’ well-being. With the projected increase in heat-related conditions due to the ongoing global increase in temperature and the aging workforce, a collaborative effort at all levels is essential and required to prevent heat stress and improve workers’ safety, health, and well-being.

## Data Availability

Some of the data presented in this study are available on request from the corresponding author due to privacy concerns.
